# Uncover the Underlying Mechanism of Drug-Induced Myopathy by Using Systems Biology Approaches

**DOI:** 10.1155/2017/9264034

**Published:** 2017-07-31

**Authors:** Dong Li, Aixin Li, Hairui Zhou, Xi Wang, Peng Li, Sheng Bi, Yang Teng

**Affiliations:** ^1^First Affiliated Hospital of Jiamusi University, Jiamusi, Heilongjiang 154002, China; ^2^Jiamusi University, Jiamusi, Heilongjiang 154002, China; ^3^Jiamusi Central Hospital, Jiamusi, Heilongjiang 154002, China

## Abstract

Drug-induced myopathy (DIM) is a rare side effect; however, the consequence could be fatal. There are few reports to systematically assess the underlying mechanism of DIM. In this study, we curated the comprehensive DIM drug list based on structured labeling products (SPLs) and carried out the analysis based on chemical structure space, drug protein interaction, side effect space, and transcriptomic profiling space. Some key features are enriched from each of analysis. Specifically, the similarity of DIM drugs is more significant than random chance, which shows that the chemical structure could distinguish the DIM-positive drugs from negatives. The cytochrome P450 (CYP) was identified to be shared by DIM drugs, which indicated the important role of metabolism in DIM. Three pathways including *pathways in cancer*, *MAPK signaling pathway*, and *GnRH signaling pathway* enriched based on transcriptomic analysis may explain the underlying mechanism of DIM. Although the DIM is the current focus of the study, the proposed approaches could be applied to other toxicity assessments and facilitate the safety evaluation.

## 1. Introduction

Myopathy is a muscular disease in which the muscle fibers do not function, resulting in muscular weakness. There are many causes for myopathy including inheritable genetic defects, metabolic disorder, exposure to toxins, and medication [1]. Although myopathies are not unusual in drug therapy, the consequence could be severe and may cause deaths. Rhabdomyolysis is a severe form of myopathy with muscle breakdown, which leads to myoglobinuria and may result in renal failure and death [2]. Different therapeutic drugs have been associated with myopathy. For example, the statin drugs which are used to lower cholesterol may cause myopathy when it is given in high dose [3]. Therefore, the US Food and Drug Administration (FDA) has recommended to limit the use of the highest approved dose of the cholesterol-lowering medication simvastatin (80 mg) because of increased risk of muscle damage such as myopathy (http://www.fda.gov/Drugs/DrugSafety/ucm256581.htm).

The mechanisms of drug-induced myopathies are complex and unknown. Metabolic change and immune-mediated disorder are two possible mechanisms [1]. Most of studies are based on case reports or focused on the certain therapeutic category. Bonifacio et al. [4] used biochemical experiments to uncover the key role of the AKT/mTOR signaling pathway in statin-induced myotoxicity. Mo et al. introduced a case report on statin-induced myopathy associated with concomitant use of cyclosporine. The patient's serum creatine kinase was significantly increased, which provides the evidence of a potential association between the elevation of creatine kinase and an increased risk of myopathy [5]. Other studies are focused on host factors such as genetic variation for drug-induced myopathy. Link et al. [6] found a strong association of myopathy with the rs4363657 single-nucleotide polymorphism (SNP) located within SLCO1B1 by using genome-wide association study (GWAS). Furthermore, it was reported that SLCO1B1 encodes the organic anion-transporting polypeptide OATP1B1, which could regulate the hepatic uptake of statins. Vicart et al. [7] reported a missense mutation in the alpha B-crystallin chaperone gene that causes a desmin-related myopathy in the French population. However, few reports provide the systematic way to assess the drug-induced myopathy (DIM), which could provide a better understanding of the underlying mechanisms of drug-induced myopathy and further develop safer drugs with low risk of myopathy.

There are two factors which hindered the deciphering of the mechanism of drug-induced myopathy—the drug itself and host information. Since the myopathy is related to the metabolic levels of individuals, it is worth investigating the genetic factor such as CYP450 in the individuals with myopathy with emerging technologies such as whole-genome sequencing (WGS) or whole-exome sequencing (WES). However, it is still hard to build the causality relationship among the genetic factors and drug taken by the patients, which leads to drug-induced myopathy. Therefore, it is necessary to investigate whether the drug properties also play a role in the drug-induced myopathy.

In this study, we hypothesized that the drug properties are associated with the cause of myopathy. The association between myopathy and the diverse of drug properties including chemical structure, side effects, protein target, and transcriptomic profiling was systematically assessed. The key features were generated to facilitate the mechanistic understanding of drug-induced myopathy.

## 2. Materials and Methods

### 2.1. Compilation of the Drug List

There are public available databases providing drug side effect data, including MetaADEDB [8] and SIDER. In this study, the SIDER database (http://sideeffects.embl.de/) was employed to extract the drugs that could cause myopathy [9]. The SIDER database consists of side effect of drugs in human, which was extracted from multiple version of structured product labeling (SPLs) by using Unified Medical Language System (UMLS) MetaMap tools [9]. In the current version (SIDER2), the database contains 996 FDA-approved drugs and 4192 side effect terms. The 4192 side effect terms were further mapped and normalized into 4500 Medical Dictionary for Regulatory Activities (MedDRA) preferred terms (PTs). We limited the term specifically to “myopathy” to obtain a drug list that causes myopathy. As a result, there are 75 drugs obtained, as shown in Supplementary Table S1 available online at https://doi.org/10.1155/2017/9264034. It is worth mentioning that we did not take into consideration of different frequency ranges in the population for side effects. Furthermore, in order to investigate the relationship between myopathy and other side effects, we composed a whole drug-side effect matrix (996 × 4500) with binary entities (1 denotes drug with side effect, otherwise 0).

### 2.2. Retrieval of Drug Properties Information

Drug structure data files (SDFs) were downloaded from PubChem database (https://pubchem.ncbi.nlm.nih.gov/) [10]. Then, the chemical descriptors were generated by using KNIME v2.5.1 (https://www.knime.org/). Specifically, the Extended Connectivity Fingerprints (ECFP-4) were used, which is well-established and extensively applied in chemical structure analysis.

The therapeutic categories of drugs were extracted from the WHO Anatomical Therapeutic Chemical (ATC) Classification System (http://www.whocc.no/atc_ddd_index/). The ATC code is a hieratical ontology structure with five levels. Naïve Bayesian classifier was used to assess the disproportionality of a specific ADR drug combination against the ADR distribution for all drugs in the global ADR database. In this study, we used the second level of the code that indicated the therapeutic main group. The details of drug profiles and side effect information were listed in Supplementary Table S1.

Drug protein target information was extracted from the DrugBank (version 4.3) database (http://www.drugbank.ca/) [11]. The target information in DrugBank was divided into four categories including therapeutic targets, enzymes, transporters, and carriers. In this study, only the protein targets in *Homo sapiens* species were employed.

### 2.3. Drug Transcriptomic Profiling Data

Transcriptomic profiles of the drugs were obtained from the Connectivity Map (version 02) generated by the Broad Institute of MIT (http://www.broadinstitute.org/cmap/) [12]. The Connectivity Map contains a collection of genome-wide transcriptional expression data of 1309 drugs from different cultured human cancer cells treated using Affymetrix Human Genome U133A 2.0 arrays. Since the array data are from different cancer cell lines, we used the prototype ranked list (PRL) by merging all the ranked lists referring to the same compound from different cell lines by using the Borda merging method [13]. Then, for each compound, the top 100 and down 100 regulated genes from PRL were considered as signature genes. Finally, we ranked the signature genes based on the frequency of myopathy drugs involved. The top 100 genes were used as the representative genes for drug-induced myopathy for further analysis.

### 2.4. Functional Analysis

Two types of functional analysis were used to interpret 100 drug-induced myopathy representative genes. First, the KEGG pathway analysis was performed to identify the significant pathways for 100 drug-induced myopathy representative genes with Fisher's exact test with multiple testing corrections by using DAVID tool (https://david.ncifcrf.gov/) [14]. Here, the signature pathways were considered with Benjamini-Hochberg (BH) adjusted *p* value less than 0.05. Then, the 100 drug-induced myopathy representative genes were mapped into a protein-protein interaction network to investigate the physical connection of genes and their functional similarity. In detail, the STRING 9.1 version [15] was applied to study protein-protein-interaction (PPI) using 100 drug-induced myopathy representative genes as input, and PPI were considered with confidence scores more than 0.4.

### 2.5. Myopathy-Related Side Effects

In order to identify the significant myopathy and related side effects, the Fisher's exact test and seven confusion matrices were generated based on drugs related to myopathy and other side effect information as described in [16] and the formulas as follows:

**Table:** 

		Side effect	
Yes	No
Myopathy	Yes	TP	TN
	No	FP	FN

(1) $$\begin{array}{c} p=\frac{\left(\begin{array}{c} TP+FN\\ TP \end{array}\right)\left(\begin{array}{c} FP+TN\\ FP \end{array}\right)}{\begin{array}{c} TP+FN+FP+TN\\ TP+FP \end{array}},\\ Accuracy=\frac{TP+TN}{TP+TN+FP+FN},\\ Sensitivity=\frac{TP}{TP+FN},\\ Specificity=\frac{TN}{TN+FP},\\ MCC=\frac{TP\times TN-FP\times FN}{\sqrt{\left(TP+FP\right)\times \left(TP+FN\right)\times \left(TN+FP\right)\times \left(TN+FN\right)}},\\ AUC=\frac{Sensitivity+Specificity}{2},\\ PPV=\frac{TP}{TP+FP},\\ NVP=\frac{TN}{FN+TN}, \end{array}$$

where TP (true positive) represents a list of drugs with investigated side effect and myopathy, FP (false positive) means a list drugs with investigated side effect but without the myopathy, TN (true negative) denotes a list of drugs not belong to investigated side effect but belong to myopathy, and FN (false negative) represents drugs neither involved in investigated side effect nor involved in myopathy.

For Fisher's exact test, the two-side *p* value was used with multiple testing corrections. As a result, significant genotype/phenotype and ADR (adverse drug reaction) pair was considered if the corrected *p* value <0.05|MCC ≥ 0.2|Sen ≥ 0.70|TP > 2. The threshold for MCC and sensitivity was decided by using empirical probability distribution function (pdf) of all the possible pairs of association; specifically, the significant thresholds located in the upper bound of the 5% quantile of the empirical pdf. The TP was decided by the consideration of the statistical power of measure, which is even high than the similar approaches [16].

### 2.6. Similarity of Myopathy Drugs

The chemical structure similarity was assessed based on ECFP-4 descriptors by using the Jaccard similarity coefficient, as shown as follows:

(2) $$\begin{array}{c} J\left({drug}_{i},{drug}_{j}\right)=\frac{{drug}_{i}\cap {drug}_{j}}{{drug}_{i}\cup {drug}_{j}}. \end{array}$$

Here, the similar drug pairs were extracted with the Jaccard similarity coefficient more than 0.4.

Furthermore, the myopathy drug similarity was assessed based on their shared protein target and distance in the PPI interaction network.

## 3. Results

**Figure 1:**
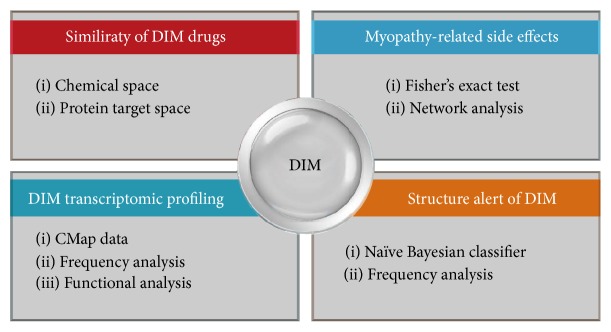
Flowchart of the study: (1) similarity between DIM drugs based on chemical similarity and protein distance in PPI network; (2) myopathy-related side effects based on Fisher's exact test and network analysis; (3) the DIM transcriptomic analysis based on CMap data; (4) structure alert of DIM.

The workflow of this study is illustrated in Figure 1. In order to systematically assess the underlying mechanisms of drug-induced myopathy, four types of analysis were carried out including (1) similarity among of DIM drugs, (2) myopathy-associated side effects, (3) transcriptomic analysis of DIM drugs and their involved pathways, and (4) structure alert of drug-induced myopathy.

### 3.1. Similarity among the DIM Drugs

**Figure 2:**
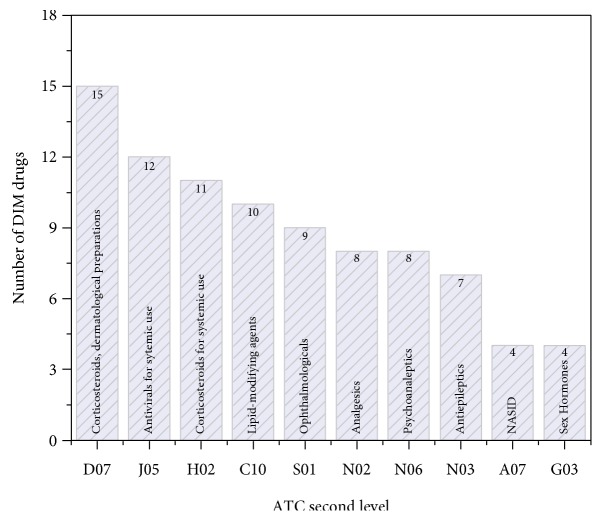
The therapeutic categories distribution of drug-induced myopathy (DIM) drugs. The DIM drugs were mapped to the second level of the WHO Anatomical Therapeutic Chemical (ATC) Classification System. Then, for each therapeutic category, the number of DIM drugs were counted.

There are a total of 75 DIM drug extracted from SIDER2 database. The distribution of DIM drugs involved therapeutic categories were shown in Figure 2. As shown in Figure 2, corticosteroids drugs such as statin are more susceptible to myopathy than other therapeutic categories. It is consistent with current observation that statin drugs were more associated with myopathy [17]. In addition, central nervous system (CNS) agents (*N02*, *N06*, and *N03*) are also needed to be cared for myopathy risk. Especially, CNS drugs are taken in high dose.

**Figure 3:**
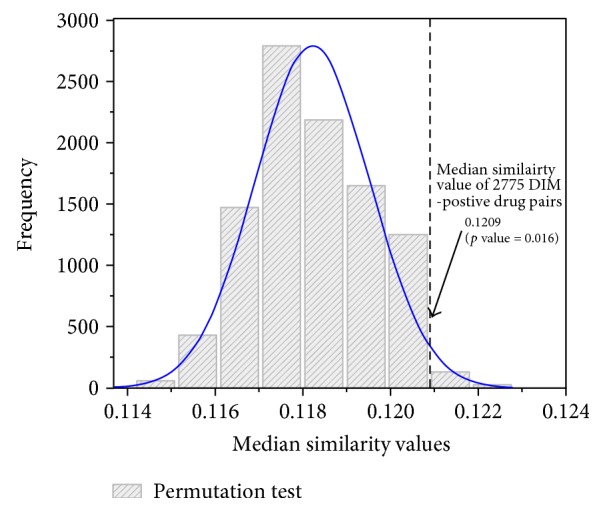
Permutation test for similarity of DIM drug pairs. The distribution of 75 DIM-positive drugs were drawn based on their chemical similarity. Then, the random test was carried out based on chemical similarity of negative DIM drugs, which are randomly picked up for 100,000 times. Then, the *p* value could be calculated for assessing whether the DIM-positive drugs are more similar.

In order to investigate whether the DIM-positive drugs share more commonality than DIM-negative drugs, we carried out pair-wise similarity analysis based on chemical structures, which generated a total of 2775 similarity pairs for 75 DIM-positive drugs. We also randomly selected the same number of DIM-negative drugs to generate a negative control for comparison. The process was repeated for 10,000 times. For each time, the *F* test was employed to investigate whether the two lists of similarity values are statistically different. The distribution of median similarity distribution values for 10,000 times randomization test is shown in Figure 3(). The median similarity values of 2775 DIM-positive drug pairs are statistically larger than those of 100,000 permutation test results (Figure 3()). It is demonstrated that the DIM drugs have some chemical structure similarity that could be used for differentiation from DIM-negative drugs.

**Figure 4:**
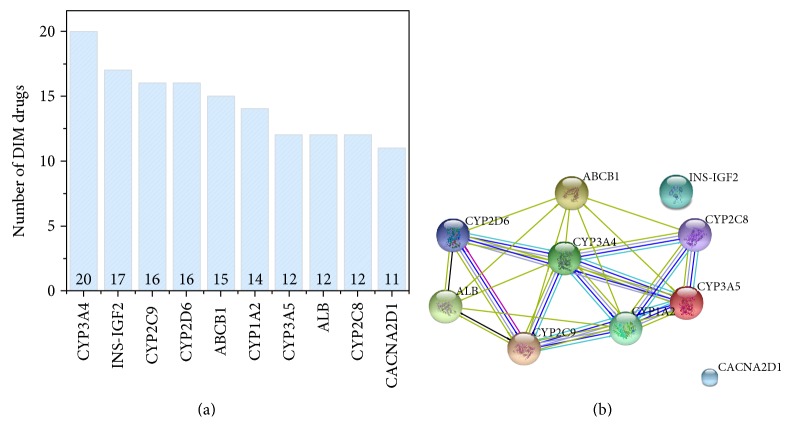
The protein space of DIM drugs. (a) The top 10 protein targets for DIM; the DIM drug and target relationship was extracted from DrugBank. (b) The STRING PPI for the top 10 protein targets; the top 10 proteins corresponding to more DIM drugs were inputted to the STRING PPI database to exact the subnetwork among the 10 proteins.

In addition, we investigated the common targets among the 75 DIM drugs.  Figure 4(a) lists the top 10 targets that are most frequently interacting with DIM drugs. It could be seen that the cytochrome P450s family dominated, indicating that the metabolism is playing a crucial role in the mechanism of DIM. It was reported that statin drugs that usually cause myopathy through statin metabolism via the CYP system [18]. Then, we mapped the 10 protein targets into STRING 9.3 PPI network to investigate whether they are interacted with each other (Figure 4(b)). It was observed that the 8 of 10 protein targets are strongly interacting with each other, which shows the similar underlying mechanism of DIM.

### 3.2. Myopathy-Associated Side Effects

**Figure 5:**
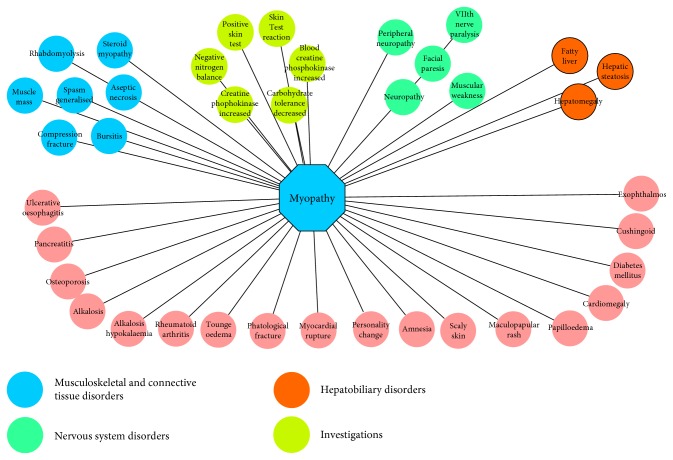
Network myopathy-related side effects. The myopathy and side effects were extracted based on SIDER database.

The cooccurrent side effects may indicate the similarity of mechanisms or causality relationship between each other. Therefore, we extracted myopathy-associated side effects to investigate whether they shared the same mechanism. As mentioned in Section 2, Fisher's exact test and network visualization were used to perform the side effect similarity analysis. There are 39 side effects associated with myopathy (Figure 5). We mapped the 39 side effects (PTs) into System Organ Class (SOC) level of MedDRA to extract their organ attributes. We highlighted four SOCs (i.e., musculoskeletal and connective tissue disorders, investigations, nervous system disorders, and hepatobiliary disorders) with more side effects (Figure 5). It was indicated that myopathy had multiple organ association with other side effects, which partly explained the multiple organ toxicity of certain therapeutic drugs [19].

We further conducted a literature survey in PubMed by using “myopathy” and enriched side effect to investigate whether the association generated could be verified by the independent studies (Table 1). For example, we found that a drug that could induce myopathy also tends to induce rheumatoid arthritis. Of the 86 patients with verified rheumatoid arthritis, 5.8% patients were found with peripheral myopathy. This observation suggests that there may be a common mechanism between myopathy and rheumatoid arthritis. Many independent verified studies in Table 1 were either case reports or control population studies, which is time-consuming and cost-intensive. Such results showed the applicability of our proposed approach on assessing the side effect relationship.

### 3.3. Transcriptomic Analysis of DIM Drugs

We mapped the 75 DIM to CMap version 02 and obtained 29 common DIM drugs. As mentioned in Section 2, the frequency analysis was conducted based on the merged signature for each DIM drug. Then, the top 100 genes were extracted as representative genes to carry out the functional analysis (Table S2).  Table 2 shows the enriched KEGG pathways for the 100 representative genes for DIM. Three pathways including *pathways in cancer*, *MAPK signaling pathway*, and *GnRH signaling pathway* were enriched. It was reported that the transforming growth factor-beta (TGF-*β*) superfamily includes a variety of cytokines expressed in the skeletal muscle. Members of this superfamily that are of great importance in the skeletal muscle are TGF-*β*1, mitogen-activated protein kinases (MAPKs), and myostatin [20]. Therefore, this shows that the underlying mechanism of myopathy could be related to MAPK pathways.

## 4. Discussion

Although the current drug toxicity focus in clinical trials is liver and cardiovascular toxicity, the high incidence of other organ toxicities such as myopathy was also worth studying. In this study, we employed systematic approaches to provide a landscape of drug-induced myopathy, which aims to provide the better understanding of the underlying mechanism of DIM for further development of safer drugs. Specifically, the DIM drugs are assessed based on chemical structure space, phenotypic information, and transcriptomic profiling. Some key features in each space are enriched, which could be used to develop screening assay or in silico approaches to further facilitate the research in this field.

Due to the prevalence issue, some side effects have more case reports than others. It limits the understanding due to idiosyncratic natures. Here, we assess the side effect similarity to enrich the myopathy-related side effect and further could derive the potential cause of myopathy. Furthermore, the multiple organ toxicity could be assessed by the proposed approach, which helps the understanding of toxicity from systems biology's point of views.

Some caveats are also important to mention. (i) In this study, we do not take into consideration of the host factor for drug-induced myopathy. However, it could play an important role. For instance, based on our analysis, we found that the CYP450 family proteins were enriched by DIM drugs, which could be quite different from individuals. However, considering the idiosyncratic nature of myopathy and causality relationship between drug and host, we only focus on drug sides. (ii) Transcriptomic data in this study was based on different cancer cell lines, which could be quite different with the muscle cells. Therefore, we do see the enriched pathways are mostly cancer related. (iii) Some other data types such as in vitro assay and high-content assay data could also provide some unique understanding for DIM. All these caveats will be accomplished in our future study. 

## 5. Conclusions

In summary, the proposed analysis pipeline could provide systematic approach to understand DIM. Although the DIM is the current focus of the study, the proposed approaches could be applied to other toxicity endpoints.

## Supplementary Material

Table S1: List of drugs caused mayopathy. Table S2: Top 100 genes found to be related to mayopathy.

## Figures and Tables

**Table 1:** Selected examples of myopathy and associated side effects by literature survey.

Myopathy-associated side effects	*p* value by Fisher's exact test	Notes	References
Diabetes mellitus	1.00*E*−11	In any diabetic condition, a failure to maintain healthy muscle is often observed and is termed diabetic myopathy	PMID: 24391596
Rhabdomyolysis	<1.00*E*−15	Rhabdomyolysis is a severe form of myopathy	PMID: 25991405
Creatine phosphokinase increased	7.00*E*−11	Increased dosage of cyclosporine induces myopathy with increased serum creatine kinase in an elderly patient on chronic statin therapy	PMID: 25512016
Neuropathy	<1.00*E*−15	Critically ill patients may develop muscle weakness or paralysis such as neuropathy during the course of sepsis and multiple organ failure	PMID: 15758592
Alkalosis hypokalaemia	6.11*E*−09	A case report about hypokalemia-induced myopathy as the first manifestation of primary hyperaldosteronism due to unilateral adrenal hyperplasia	PMID: 19829865
Rheumatoid arthritis	9.69*E*−09	Based on 86 patients with verified rheumatoid arthritis, 5.8% patients were found with peripheral myopathy	PMID: 13917616
Osteoporosis	3.90*E*−10	Chronic use of glucocorticoids (GCs) is the most common cause of secondary osteoporosis. The glucocorticoids induce myopathy as well	PMID: 22870429
Hepatic steatosis	7.20*E*−10	A case report showed that zidovudine treatment can induce mitochondrial multisystem disease, as revealed in our case by myopathy, liver steatosis, and lactic acidosis	PMID: 9927163
Cardiomegaly	1.60*E*−10	Two unrelated 16-year-old boys had mental retardation, cardiomegaly, and proximal myopathy	PMID: 6450334

**Table 2:** Enriched KEGG pathways for 100 representative genes.

Pathways	Number of hits	Involved genes	Adjusted *p* value
Pathways in cancer	9	Jun, Runx1, MAX, Fas, FGF22, HSP90AA2, Cdk2, ETS1, Cdc42	0.005
MAPK signaling pathway	7	Jun, MAX, Fas, FGF22, mapt, Il1r1, Cdc42	0.024
GnRH signaling pathway	4	Jun, ITPR2, Gnas, Cdc42	0.049
