# Supplementary material for: Uncover the Underlying Mechanism of Drug-Induced Myopathy by Using Systems Biology Approaches
Source: Int J Genomics. 2017 Jul 31;2017:9264034. doi: 10.1155/2017/9264034 (PMC5554993; doi:10.1155/2017/9264034)
Supplement: Supplementary file 1 — Table S1: List of drugs caused mayopathy. Table S2: Top 100 genes found to be related to mayopathy. [file 9264034.f1.pdf]

Table S1

List of drugs caused mayopathy

| ***Drug names*** | ***Drugbank ID*** | ***PubCID*** | ***SIDER database link*** | ***ATC code*** |
|---|---|---|---|---|
| zalcitabine | DB00943 | 5718 | http://sideeffects.embl.de/drugs/5718/ | J05AF03 |
| daptomycin | DB00080 | 16129629 | http://sideeffects.embl.de/drugs/16129629/ | J01XX09 |
| cyclosporine | DB00091 | 2909 | http://sideeffects.embl.de/drugs/2909/ | L04AA01 |
| acamprosate | DB00659 | 71158 | http://sideeffects.embl.de/drugs/71158/ | N07BB03 |
| allopurinol | DB00437 | 2094 | http://sideeffects.embl.de/drugs/2094/ | M04AA01 |
| almotriptan | DB00918 | 123606 | http://sideeffects.embl.de/drugs/123606/ | N02CC05 |
| amiodarone | DB01118 | 2156 | http://sideeffects.embl.de/drugs/2156/ | C01BD01 |
| aripiprazole | DB01238 | 60795 | http://sideeffects.embl.de/drugs/60795/ | N05AX12 |
| atazanavir | DB01072 | 148192 | http://sideeffects.embl.de/drugs/148192/ | J05AE08 |
| atorvastatin | DB01076 | 2250 | http://sideeffects.embl.de/drugs/2250/ | C10AA05 |
| candesartan | DB00796 | 2541 | http://sideeffects.embl.de/drugs/2541/ | C09CA06 |
| cerivastatin sodium | DB00439 | 2676 | http://sideeffects.embl.de/drugs/2676/ | C10AA06 |
| chloroquine | DB00608 | 2719 | http://sideeffects.embl.de/drugs/2719/ | P01BA01 P01BA02 |
| cisatracurium | DB00565 | 47320 | http://sideeffects.embl.de/drugs/47320/ | M03AC11 |
| clomipramine | DB01242 | 2801 | http://sideeffects.embl.de/drugs/2801/ | N06AA04 |
| colchicine | DB01394 | 2833 | http://sideeffects.embl.de/drugs/2833/ | M04AC01 |
| hydrocortisone | DB00741 | 3640 | http://sideeffects.embl.de/drugs/3640/ | A01AC03 A07EA02 C05AA01 D07AA02 |
| cortisone | DB01380 | 222786 | http://sideeffects.embl.de/drugs/222786/ | H02AB10 S01BA03 |
| betamethasone | DB01234 | 3003 | http://sideeffects.embl.de/drugs/3003/ | A01AC02 C05AA09 D07AB19 D07XB05 |
| didanosine | DB00900 | 3043 | http://sideeffects.embl.de/drugs/3043/ | J05AF02 |
| diltiazem | DB00343 | 3075 | http://sideeffects.embl.de/drugs/3075/ | C08DB01 |
| cromolyn | DB01003 | 2881 | http://sideeffects.embl.de/drugs/2881/ | R01AC01 A07EB01 S01GX01 R03BC01 |
| efavirenz | DB00625 | 3203 | http://sideeffects.embl.de/drugs/3203/ | J05AG03 |
| eletriptan | DB00216 | 77993 | http://sideeffects.embl.de/drugs/77993/ | N02CC06 |
| aminocaproic acid | DB00513 | 564 | http://sideeffects.embl.de/drugs/564/ | B02AA01 |
| ezetimibe | DB00973 | 150311 | http://sideeffects.embl.de/drugs/150311/ | C10AX09 |
| fentanyl | DB00813 | 3345 | http://sideeffects.embl.de/drugs/3345/ | N01AH01 N02AB03 |
| fludrocortisone | DB00687 | 31378 | http://sideeffects.embl.de/drugs/31378/ | H02AA02 |
| fluoxetine | DB00472 | 3386 | http://sideeffects.embl.de/drugs/3386/ | N06AB03 |
| fluvastatin | DB01095 | 3403 | http://sideeffects.embl.de/drugs/3403/ | C10AA04 |
| fluvoxamine | DB00176 | 3404 | http://sideeffects.embl.de/drugs/3404/ | N06AB08 |
| foscarnet | DB00529 | 3414 | http://sideeffects.embl.de/drugs/3414/ | J05AD01 |
| fosphenytoin | DB00252 | 56338 | http://sideeffects.embl.de/drugs/56338/ | N03AB02 N03AB04 N03AB05 |
| gemfibrozil | DB01241 | 3463 | http://sideeffects.embl.de/drugs/3463/ | C10AB04 |
| glatiramer acetate | DB05259 | 3081884 | http://sideeffects.embl.de/drugs/3081884/ | L03AX13 |
| hydroxychloroquine | DB01611 | 3652 | http://sideeffects.embl.de/drugs/3652/ | P01BA02 |
| indapamide | DB00808 | 3702 | http://sideeffects.embl.de/drugs/3702/ | C03BA11 |
| labetalol | DB00598 | 3869 | http://sideeffects.embl.de/drugs/3869/ | C07AG01 |
| lamivudine | DB00709 | 3877 | http://sideeffects.embl.de/drugs/3877/ | J05AF05 |
| leuprolide | DB00007 | 3911 | http://sideeffects.embl.de/drugs/3911/ | L02AE02 |
| lovastatin | DB00227 | 3962 | http://sideeffects.embl.de/drugs/3962/ | C10AA02 |
| methylprednisolone | DB00959 | 4159 | http://sideeffects.embl.de/drugs/4159/ | D07AA01 D07AC14 D10AA02 H02AB04 |
| nelfinavir | DB00220 | 4451 | http://sideeffects.embl.de/drugs/4451/ | J05AE04 |
| niacin | DB00627 | 937 | http://sideeffects.embl.de/drugs/937/ | C04AC01 C10AD02 |
| olanzapine | DB00334 | 4585 | http://sideeffects.embl.de/drugs/4585/ | N05AH03 |
| paroxetine | DB00715 | 4691 | http://sideeffects.embl.de/drugs/4691/ | N06AB05 |
| phenytoin | DB00252 | 1775 | http://sideeffects.embl.de/drugs/1775/ | N03AB02 N03AB04 N03AB05 |
| pramipexole | DB00413 | 4885 | http://sideeffects.embl.de/drugs/4885/ | N04BC05 |
| pravastatin | DB00175 | 4889 | http://sideeffects.embl.de/drugs/4889/ | C10AA03 |
| prednisolone | DB00860 | 4894 | http://sideeffects.embl.de/drugs/4894/ | A07EA01 C05AA04 D07AA01 D07AA03 D07AC14 D07XA02 D10AA02 H02AB04 |
| prednisone | DB00635 | 4900 | http://sideeffects.embl.de/drugs/4900/ | A07EA03 H02AB07 H02AB15 |
| pregabalin | DB00230 | 125889 | http://sideeffects.embl.de/drugs/125889/ | N03AX16 |
| progesterone | DB00396 | 4920 | http://sideeffects.embl.de/drugs/4920/ | G03AC06 G03DA02 G03DA03 G03DA04 |
| propoxyphene | DB00647 | 10100 | http://sideeffects.embl.de/drugs/10100/ | N02AC04 N02AC54 N02AC74 |
| raltegravir | NA | 11598201 | http://sideeffects.embl.de/drugs/11598201/ | J05AX08 |
| rifampin | DB01045 | 5381226 | http://sideeffects.embl.de/drugs/5381226/ | J04AB02 |
| rocuronium | DB00728 | 60695 | http://sideeffects.embl.de/drugs/60695/ | M03AC09 |
| rofecoxib | DB00533 | 5090 | http://sideeffects.embl.de/drugs/5090/ | M01AH02 |
| rosuvastatin | DB01098 | 446157 | http://sideeffects.embl.de/drugs/446157/ | C10AA07 |
| saquinavir | DB01232 | 60787 | http://sideeffects.embl.de/drugs/60787/ | J05AE01 |
| selegiline | DB01037 | 5195 | http://sideeffects.embl.de/drugs/5195/ | N04BD01 |
| simvastatin | DB00641 | 54454 | http://sideeffects.embl.de/drugs/54454/ | C10AA01 |
| sumatriptan | DB00669 | 5358 | http://sideeffects.embl.de/drugs/5358/ | N02CC01 |
| sunitinib | DB01268 | 3086686 | http://sideeffects.embl.de/drugs/3086686/ | L01XE04 |
| tacrine | DB00382 | 1935 | http://sideeffects.embl.de/drugs/1935/ | N06AA18 N06DA01 |
| temozolomide | DB00853 | 5394 | http://sideeffects.embl.de/drugs/5394/ | L01AX03 |
| viread | DB00300 | 119830 | http://sideeffects.embl.de/drugs/119830/ | J05AF07 |

| | | | | |
|---|---|---|---|---|
| telbivudine | DB01265 | 1134 | http://sideeffects.embl.de/drugs/1134/ | J05AF11 |
| tramadol | DB00193 | 5523 | http://sideeffects.embl.de/drugs/5523/ | N02AX02 |
| triamcinolone | DB00620 | 5544 | http://sideeffects.embl.de/drugs/5544/ | A01AC01 D07AB09 D07XB02 H02AB08 |
| venlafaxine | DB00285 | 5656 | http://sideeffects.embl.de/drugs/5656/ | N06AX16 N06AX23 |
| voriconazole | DB00582 | 71616 | http://sideeffects.embl.de/drugs/71616/ | J02AC03 |
| zidovudine | DB00495 | 5726 | http://sideeffects.embl.de/drugs/5726/ | J05AF01 |
| ziprasidone | DB00246 | 60853 | http://sideeffects.embl.de/drugs/60853/ | N05AE04 |
| eszopiclone | DB01198 | 5735 | http://sideeffects.embl.de/drugs/5735/ | N05CF01 |

Table S2

Top 100 genes found to be related to mayopathy

| From | To | Species | Gene Name |
|---|---|---|---|
| 8880 | FUBP1 | Homo sapiens | far upstream element (FUSE) binding protein 1 |
| 3725 | Jun | Homo sapiens | jun oncogene |
| 3872 | KRT17P3 | Homo sapiens | keratin 17; keratin 17 pseudogene 3 |
| 3872 | KRT17 | Homo sapiens | keratin 17; keratin 17 pseudogene 3 |
| 2099 | Esr1 | Homo sapiens | estrogen receptor 1 |
| 3535 | IGLV2-11 | Homo sapiens | immunoglobulin lambda variable 2-11; immunoglobulin lambda constant 2 (Kern-Oz- marker); immunoglobulin lambda variable 1-44; immunoglobulin lambda constant 1 (Mcg marker); immunoglobulin lambda variable 1-40; immunoglobulin lambda variable 3-21; immunoglobulin lambda locus; immunoglobulin lambda constant 3 (Kern-Oz+ marker) |
| 3535 | IGLV1-40 | Homo sapiens | immunoglobulin lambda variable 2-11; immunoglobulin lambda constant 2 (Kern-Oz- marker); immunoglobulin lambda variable 1-44; immunoglobulin lambda constant 1 (Mcg marker); immunoglobulin lambda variable 1-40; immunoglobulin lambda variable 3-21; immunoglobulin lambda locus; immunoglobulin lambda constant 3 (Kern-Oz+ marker) |
| 3535 | IGLV1-44 | Homo sapiens | immunoglobulin lambda variable 2-11; immunoglobulin lambda constant 2 (Kern-Oz- marker); immunoglobulin lambda variable 1-44; immunoglobulin lambda constant 1 (Mcg marker); immunoglobulin lambda variable 1-40; immunoglobulin lambda variable 3-21; immunoglobulin lambda locus; immunoglobulin lambda constant 3 (Kern-Oz+ marker) |
| 3535 | IGL@ | Homo sapiens | immunoglobulin lambda variable 2-11; immunoglobulin lambda constant 2 (Kern-Oz- marker); immunoglobulin lambda variable 1-44; immunoglobulin lambda constant 1 (Mcg marker); immunoglobulin lambda variable 1-40; immunoglobulin lambda variable 3-21; immunoglobulin lambda locus; immunoglobulin lambda constant 3 (Kern-Oz+ marker) |
| 3535 | iglC1 | Homo sapiens | immunoglobulin lambda variable 2-11; immunoglobulin lambda constant 2 (Kern-Oz- marker); immunoglobulin lambda variable 1-44; immunoglobulin lambda constant 1 (Mcg marker); immunoglobulin lambda variable 1-40; immunoglobulin lambda variable 3-21; immunoglobulin lambda locus; immunoglobulin lambda constant 3 (Kern-Oz+ marker) |
| 3535 | iglC2 | Homo sapiens | immunoglobulin lambda variable 2-11; immunoglobulin lambda constant 2 (Kern-Oz- marker); immunoglobulin lambda variable 1-44; immunoglobulin lambda constant 1 (Mcg marker); immunoglobulin lambda variable 1-40; immunoglobulin lambda variable 3-21; immunoglobulin lambda locus; immunoglobulin lambda constant 3 (Kern-Oz+ marker) |
| 3535 | IGLV3-21 | Homo sapiens | immunoglobulin lambda variable 2-11; immunoglobulin lambda constant 2 (Kern-Oz- marker); immunoglobulin lambda variable 1-44; immunoglobulin lambda constant 1 (Mcg marker); immunoglobulin lambda variable 1-40; immunoglobulin lambda variable 3-21; immunoglobulin lambda locus; immunoglobulin lambda constant 3 (Kern-Oz+ marker) |
| 3535 | IGLC3 | Homo sapiens | immunoglobulin lambda variable 2-11; immunoglobulin lambda constant 2 (Kern-Oz- marker); immunoglobulin lambda variable 1-44; immunoglobulin lambda constant 1 (Mcg marker); immunoglobulin lambda variable 1-40; immunoglobulin lambda variable 3-21; immunoglobulin lambda locus; immunoglobulin lambda constant 3 (Kern-Oz+ marker) |
| 4929 | NR4A2 | Homo sapiens | nuclear receptor subfamily 4, group A, member 2 |
| 414062 | CCL3L1 | Homo sapiens | chemokine (C-C motif) ligand 3-like 3; chemokine (C-C motif) ligand 3-like 1 |
| 414062 | CCL3L3 | Homo sapiens | chemokine (C-C motif) ligand 3-like 3; chemokine (C-C motif) ligand 3-like 1 |
| 1289 | Col5a1 | Homo sapiens | collagen, type V, alpha 1 |
| 5229 | Pggt1b | Homo sapiens | protein geranylgeranyltransferase type I, beta subunit |
| 26707 | OR2J2 | Homo sapiens | olfactory receptor, family 2, subfamily J, member 2 |
| 5178 | PEG3AS | Homo sapiens | paternally expressed 3; PEG3 antisense RNA (non-protein coding); zinc finger, imprinted 2 |
| 5178 | ZIM2 | Homo sapiens | paternally expressed 3; PEG3 antisense RNA (non-protein coding); zinc finger, imprinted 2 |
| 5178 | PEG3 | Homo sapiens | paternally expressed 3; PEG3 antisense RNA (non-protein coding); zinc finger, imprinted 2 |

| | | | |
|---|---|---|---|
| 26156 | Rsl1d1 | Homo sapiens | ribosomal L1 domain containing 1 |
| 6299 | sall1 | Homo sapiens | sal-like 1 (Drosophila) |
| 1080 | CFTR | Homo sapiens | cystic fibrosis transmembrane conductance regulator (ATP-binding cassette sub-family C, member 7) |
| 1896 | eda | Homo sapiens | ectodysplasin A |
| 4116 | MAGOH | Homo sapiens | mago-nashi homolog, proliferation-associated (Drosophila) |
| 7940 | Lst1 | Homo sapiens | leukocyte specific transcript 1 |
| 3856 | KRT8P9 | Homo sapiens | keratin 8 pseudogene 9; similar to keratin 8; keratin 8 |
| 3856 | LOC149501 | Homo sapiens | keratin 8 pseudogene 9; similar to keratin 8; keratin 8 |
| 3856 | krt8 | Homo sapiens | keratin 8 pseudogene 9; similar to keratin 8; keratin 8 |
| 6974 | TRGV2 | Homo sapiens | T cell receptor gamma variable 2 |
| 23063 | WAPAL | Homo sapiens | wings apart-like homolog (Drosophila) |
| 1519 | Ctso | Homo sapiens | cathepsin O |
| 1910 | ednrb | Homo sapiens | endothelin receptor type B |
| 23530 | Nnt | Homo sapiens | nicotinamide nucleotide transhydrogenase |
| 2314 | fliI | Homo sapiens | flightless I homolog (Drosophila) |
| 821 | Canx | Homo sapiens | calnexin |
| 23197 | FAF2 | Homo sapiens | Fas associated factor family member 2 |
| 2273 | fhl1 | Homo sapiens | four and a half LIM domains 1 |
| 5564 | PRKAB1 | Homo sapiens | protein kinase, AMP-activated, beta 1 non-catalytic subunit |
| 3320 | HSP90AA2 | Homo sapiens | heat shock protein 90kDa alpha (cytosolic), class A member 2; heat shock protein 90kDa alpha (cytosolic), class A member 1 |
| 3320 | HSP90AA1 | Homo sapiens | heat shock protein 90kDa alpha (cytosolic), class A member 2; heat shock protein 90kDa alpha (cytosolic), class A member 1 |
| 1462 | VCAN | Homo sapiens | versican |
| 4017 | loxl2 | Homo sapiens | lysyl oxidase-like 2 |
| 5050 | PAFAH1B3 | Homo sapiens | platelet-activating factor acetylhydrolase, isoform Ib, subunit 3 (29kDa) |
| 27006 | FGF22 | Homo sapiens | fibroblast growth factor 22 |
| 27005 | USP21 | Homo sapiens | ubiquitin specific peptidase 21 |
| 3119 | HLA-DQB1 | Homo sapiens | major histocompatibility complex, class II, DQ beta 1; similar to major histocompatibility complex, class II, DQ beta 1 |
| 3119 | LOC100133583 | Homo sapiens | major histocompatibility complex, class II, DQ beta 1; similar to major histocompatibility complex, class II, DQ beta 1 |
| 286451 | YIPF6 | Homo sapiens | Yip1 domain family, member 6 |
| 2778 | Gnas | Homo sapiens | GNAS complex locus |
| 5621 | PRNP | Homo sapiens | prion protein |
| 7430 | EZR | Homo sapiens | hypothetical protein LOC100129652; ezrin |
| 7430 | LOC100129652 | Homo sapiens | hypothetical protein LOC100129652; ezrin |
| 9229 | dlgap1 | Homo sapiens | discs, large (Drosophila) homolog-associated protein 1 |
| 114884 | OSBPL10 | Homo sapiens | oxysterol binding protein-like 10 |
| 3425 | Idua | Homo sapiens | iduronidase, alpha-L- |
| 6382 | sdc1 | Homo sapiens | syndecan 1 |
| 4772 | nfatc1 | Homo sapiens | nuclear factor of activated T-cells, cytoplasmic, calcineurin-dependent 1 |
| 998 | Cdc42 | Homo sapiens | cell division cycle 42 (GTP binding protein, 25kDa); cell division cycle 42 pseudogene 2 |
| 998 | CDC42P2 | Homo sapiens | cell division cycle 42 (GTP binding protein, 25kDa); cell division cycle 42 pseudogene 2 |
| 4149 | MAX | Homo sapiens | MYC associated factor X |
| 4147 | Matn2 | Homo sapiens | matrilin 2 |
| 7026 | NR2F2 | Homo sapiens | nuclear receptor subfamily 2, group F, member 2 |
| 1937 | EEF1G | Homo sapiens | eukaryotic translation elongation factor 1 gamma |
| 8572 | PDLIM4 | Homo sapiens | PDZ and LIM domain 4 |
| 54454 | atad2b | Homo sapiens | ATPase family, AAA domain containing 2B |
| 5912 | RAP2B | Homo sapiens | RAP2B, member of RAS oncogene family |
| 27239 | GPR162 | Homo sapiens | G protein-coupled receptor 162 |
| 7991 | TUSC3 | Homo sapiens | tumor suppressor candidate 3 |
| 2192 | FBLN1 | Homo sapiens | fibulin 1 |
| 3964 | LGALS8 | Homo sapiens | lectin, galactoside-binding, soluble, 8 |

| | | | |
|---|---|---|---|
| 6929 | tcf3 | Homo sapiens | transcription factor 3 (E2A immunoglobulin enhancer binding factors E12/E47) |
| 2575 | GAGE2A | Homo sapiens | G antigen 2A; G antigen 2B; G antigen 12I; G antigen 12F; G antigen 2E; G antigen 12G; G antigen 12D; G antigen 1; G antigen 2C; G antigen 12E; G antigen 2D; G antigen 12B; G antigen 3; G antigen 4; G antigen 12C; G antigen 5; G antigen 6; G antigen 7; G antigen 8 |
| 2575 | GAGE2D | Homo sapiens | G antigen 2A; G antigen 2B; G antigen 12I; G antigen 12F; G antigen 2E; G antigen 12G; G antigen 12D; G antigen 1; G antigen 2C; G antigen 12E; G antigen 2D; G antigen 12B; G antigen 3; G antigen 4; G antigen 12C; G antigen 5; G antigen 6; G antigen 7; G antigen 8 |
| 2575 | GAGE2E | Homo sapiens | G antigen 2A; G antigen 2B; G antigen 12I; G antigen 12F; G antigen 2E; G antigen 12G; G antigen 12D; G antigen 1; G antigen 2C; G antigen 12E; G antigen 2D; G antigen 12B; G antigen 3; G antigen 4; G antigen 12C; G antigen 5; G antigen 6; G antigen 7; G antigen 8 |
| 2575 | GAGE12G | Homo sapiens | G antigen 2A; G antigen 2B; G antigen 12I; G antigen 12F; G antigen 2E; G antigen 12G; G antigen 12D; G antigen 1; G antigen 2C; G antigen 12E; G antigen 2D; G antigen 12B; G antigen 3; G antigen 4; G antigen 12C; G antigen 5; G antigen 6; G antigen 7; G antigen 8 |
| 2575 | GAGE12F | Homo sapiens | G antigen 2A; G antigen 2B; G antigen 12I; G antigen 12F; G antigen 2E; G antigen 12G; G antigen 12D; G antigen 1; G antigen 2C; G antigen 12E; G antigen 2D; G antigen 12B; G antigen 3; G antigen 4; G antigen 12C; G antigen 5; G antigen 6; G antigen 7; G antigen 8 |
| 2575 | GAGE2B | Homo sapiens | G antigen 2A; G antigen 2B; G antigen 12I; G antigen 12F; G antigen 2E; G antigen 12G; G antigen 12D; G antigen 1; G antigen 2C; G antigen 12E; G antigen 2D; G antigen 12B; G antigen 3; G antigen 4; G antigen 12C; G antigen 5; G antigen 6; G antigen 7; G antigen 8 |
| 2575 | GAGE12E | Homo sapiens | G antigen 2A; G antigen 2B; G antigen 12I; G antigen 12F; G antigen 2E; G antigen 12G; G antigen 12D; G antigen 1; G antigen 2C; G antigen 12E; G antigen 2D; G antigen 12B; G antigen 3; G antigen 4; G antigen 12C; G antigen 5; G antigen 6; G antigen 7; G antigen 8 |
| 2575 | GAGE2C | Homo sapiens | G antigen 2A; G antigen 2B; G antigen 12I; G antigen 12F; G antigen 2E; G antigen 12G; G antigen 12D; G antigen 1; G antigen 2C; G antigen 12E; G antigen 2D; G antigen 12B; G antigen 3; G antigen 4; G antigen 12C; G antigen 5; G antigen 6; G antigen 7; G antigen 8 |
| 2575 | GAGE12D | Homo sapiens | G antigen 2A; G antigen 2B; G antigen 12I; G antigen 12F; G antigen 2E; G antigen 12G; G antigen 12D; G antigen 1; G antigen 2C; G antigen 12E; G antigen 2D; G antigen 12B; G antigen 3; G antigen 4; G antigen 12C; G antigen 5; G antigen 6; G antigen 7; G antigen 8 |
| 2575 | GAGE12C | Homo sapiens | G antigen 2A; G antigen 2B; G antigen 12I; G antigen 12F; G antigen 2E; G antigen 12G; G antigen 12D; G antigen 1; G antigen 2C; G antigen 12E; G antigen 2D; G antigen 12B; G antigen 3; G antigen 4; G antigen 12C; G antigen 5; G antigen 6; G antigen 7; G antigen 8 |
| 2575 | GAGE12B | Homo sapiens | G antigen 2A; G antigen 2B; G antigen 12I; G antigen 12F; G antigen 2E; G antigen 12G; G antigen 12D; G antigen 1; G antigen 2C; G antigen 12E; G antigen 2D; G antigen 12B; G antigen 3; G antigen 4; G antigen 12C; G antigen 5; G antigen 6; G antigen 7; G antigen 8 |
| 2575 | GAGE1 | Homo sapiens | G antigen 2A; G antigen 2B; G antigen 12I; G antigen 12F; G antigen 2E; G antigen 12G; G antigen 12D; G antigen 1; G antigen 2C; G antigen 12E; G antigen 2D; G antigen 12B; G antigen 3; G antigen 4; G antigen 12C; G antigen 5; G antigen 6; G antigen 7; G antigen 8 |
| 2575 | GAGE3 | Homo sapiens | G antigen 2A; G antigen 2B; G antigen 12I; G antigen 12F; G antigen 2E; G antigen 12G; G antigen 12D; G antigen 1; G antigen 2C; G antigen 12E; G antigen 2D; G antigen 12B; G antigen 3; G antigen 4; G antigen 12C; G antigen 5; G antigen 6; G antigen 7; G antigen 8 |
| 2575 | GAGE5 | Homo sapiens | G antigen 2A; G antigen 2B; G antigen 12I; G antigen 12F; G antigen 2E; G antigen 12G; G antigen 12D; G antigen 1; G antigen 2C; G antigen 12E; G antigen 2D; G antigen 12B; G antigen 3; G antigen 4; G antigen 12C; G antigen 5; G antigen 6; G antigen 7; G antigen 8 |

| | | | |
|---|---|---|---|
| 2575 | GAGE4 | Homo sapiens | G antigen 2A; G antigen 2B; G antigen 12I; G antigen 12F; G antigen 2E; G antigen 12G; G antigen 12D; G antigen 1; G antigen 2C; G antigen 12E; G antigen 2D; G antigen 12B; G antigen 3; G antigen 4; G antigen 12C; G antigen 5; G antigen 6; G antigen 7; G antigen 8 |
| 2575 | GAGE7 | Homo sapiens | G antigen 2A; G antigen 2B; G antigen 12I; G antigen 12F; G antigen 2E; G antigen 12G; G antigen 12D; G antigen 1; G antigen 2C; G antigen 12E; G antigen 2D; G antigen 12B; G antigen 3; G antigen 4; G antigen 12C; G antigen 5; G antigen 6; G antigen 7; G antigen 8 |
| 2575 | GAGE12I | Homo sapiens | G antigen 2A; G antigen 2B; G antigen 12I; G antigen 12F; G antigen 2E; G antigen 12G; G antigen 12D; G antigen 1; G antigen 2C; G antigen 12E; G antigen 2D; G antigen 12B; G antigen 3; G antigen 4; G antigen 12C; G antigen 5; G antigen 6; G antigen 7; G antigen 8 |
| 2575 | GAGE6 | Homo sapiens | G antigen 2A; G antigen 2B; G antigen 12I; G antigen 12F; G antigen 2E; G antigen 12G; G antigen 12D; G antigen 1; G antigen 2C; G antigen 12E; G antigen 2D; G antigen 12B; G antigen 3; G antigen 4; G antigen 12C; G antigen 5; G antigen 6; G antigen 7; G antigen 8 |
| 2575 | GAGE8 | Homo sapiens | G antigen 2A; G antigen 2B; G antigen 12I; G antigen 12F; G antigen 2E; G antigen 12G; G antigen 12D; G antigen 1; G antigen 2C; G antigen 12E; G antigen 2D; G antigen 12B; G antigen 3; G antigen 4; G antigen 12C; G antigen 5; G antigen 6; G antigen 7; G antigen 8 |
| 780 | DDR1 | Homo sapiens | discoidin domain receptor tyrosine kinase 1 |
| 1137 | Chrna4 | Homo sapiens | cholinergic receptor, nicotinic, alpha 4 |
| 9612 | ncor2 | Homo sapiens | nuclear receptor co-repressor 2 |
| 6738 | Trove2 | Homo sapiens | TROVE domain family, member 2 |
| 2618 | GART | Homo sapiens | phosphoribosylglycinamide formyltransferase, phosphoribosylglycinamide synthetase, phosphoribosylaminoimidazole synthetase |
| 2262 | Gpc5 | Homo sapiens | glypican 5 |
| 55612 | FERMT1 | Homo sapiens | fermitin family homolog 1 (Drosophila) |
| 2113 | ETS1 | Homo sapiens | v-ets erythroblastosis virus E26 oncogene homolog 1 (avian) |
| 9788 | mtss1 | Homo sapiens | metastasis suppressor 1 |
| 4162 | MCAM | Homo sapiens | melanoma cell adhesion molecule |
| 4168 | MCF2 | Homo sapiens | MCF.2 cell line derived transforming sequence |
| 861 | Runx1 | Homo sapiens | runt-related transcription factor 1 |
| 3500 | IGHV3-11 | Homo sapiens | immunoglobulin heavy constant gamma 1 (G1m marker); immunoglobulin heavy constant mu; immunoglobulin heavy variable 3-7; immunoglobulin heavy constant gamma 3 (G3m marker); immunoglobulin heavy variable 3-11 (gene/pseudogene); immunoglobulin heavy variable 4-31; immunoglobulin heavy locus |
| 3500 | IGHV3-7 | Homo sapiens | immunoglobulin heavy constant gamma 1 (G1m marker); immunoglobulin heavy constant mu; immunoglobulin heavy variable 3-7; immunoglobulin heavy constant gamma 3 (G3m marker); immunoglobulin heavy variable 3-11 (gene/pseudogene); immunoglobulin heavy variable 4-31; immunoglobulin heavy locus |
| 3500 | IGHG3 | Homo sapiens | immunoglobulin heavy constant gamma 1 (G1m marker); immunoglobulin heavy constant mu; immunoglobulin heavy variable 3-7; immunoglobulin heavy constant gamma 3 (G3m marker); immunoglobulin heavy variable 3-11 (gene/pseudogene); immunoglobulin heavy variable 4-31; immunoglobulin heavy locus |
| 3500 | Ighg1 | Homo sapiens | immunoglobulin heavy constant gamma 1 (G1m marker); immunoglobulin heavy constant mu; immunoglobulin heavy variable 3-7; immunoglobulin heavy constant gamma 3 (G3m marker); immunoglobulin heavy variable 3-11 (gene/pseudogene); immunoglobulin heavy variable 4-31; immunoglobulin heavy locus |
| 3500 | IGH@ | Homo sapiens | immunoglobulin heavy constant gamma 1 (G1m marker); immunoglobulin heavy constant mu; immunoglobulin heavy variable 3-7; immunoglobulin heavy constant gamma 3 (G3m marker); immunoglobulin heavy variable 3-11 (gene/pseudogene); immunoglobulin heavy variable 4-31; immunoglobulin heavy locus |
| 3500 | IGHM | Homo sapiens | immunoglobulin heavy constant gamma 1 (G1m marker); immunoglobulin heavy constant mu; immunoglobulin heavy variable 3-7; immunoglobulin heavy constant gamma 3 (G3m marker); immunoglobulin heavy variable 3-11 (gene/pseudogene); immunoglobulin heavy variable 4-31; immunoglobulin heavy locus |

| | | | |
|---|---|---|---|
| 3500 | ighv4-31 | Homo sapiens | immunoglobulin heavy constant gamma 1 (G1m marker); immunoglobulin heavy constant mu; immunoglobulin heavy variable 3-7; immunoglobulin heavy constant gamma 3 (G3m marker); immunoglobulin heavy variable 3-11 (gene/pseudogene); immunoglobulin heavy variable 4-31; immunoglobulin heavy locus |
| 3705 | ITPK1 | Homo sapiens | inositol 1,3,4-triphosphate 5/6 kinase |
| 606500 | SNORD68 | Homo sapiens | small nucleolar RNA, C/D box 68 |
| 4128 | MAOA | Homo sapiens | monoamine oxidase A |
| 5087 | PBX1 | Homo sapiens | pre-B-cell leukemia homeobox 1 |
| 7001 | PRDX2 | Homo sapiens | peroxiredoxin 2 |
| 3709 | ITPR2 | Homo sapiens | inositol 1,4,5-triphosphate receptor, type 2 |
| 5662 | psd | Homo sapiens | pleckstrin and Sec7 domain containing |
| 3507 | IGHV3-11 | Homo sapiens | immunoglobulin heavy constant gamma 1 (G1m marker); immunoglobulin heavy constant mu; immunoglobulin heavy variable 3-7; immunoglobulin heavy constant gamma 3 (G3m marker); immunoglobulin heavy variable 3-11 (gene/pseudogene); immunoglobulin heavy variable 4-31; immunoglobulin heavy locus |
| 3507 | IGHV3-7 | Homo sapiens | immunoglobulin heavy constant gamma 1 (G1m marker); immunoglobulin heavy constant mu; immunoglobulin heavy variable 3-7; immunoglobulin heavy constant gamma 3 (G3m marker); immunoglobulin heavy variable 3-11 (gene/pseudogene); immunoglobulin heavy variable 4-31; immunoglobulin heavy locus |
| 3507 | IGHG3 | Homo sapiens | immunoglobulin heavy constant gamma 1 (G1m marker); immunoglobulin heavy constant mu; immunoglobulin heavy variable 3-7; immunoglobulin heavy constant gamma 3 (G3m marker); immunoglobulin heavy variable 3-11 (gene/pseudogene); immunoglobulin heavy variable 4-31; immunoglobulin heavy locus |
| 3507 | Ighg1 | Homo sapiens | immunoglobulin heavy constant gamma 1 (G1m marker); immunoglobulin heavy constant mu; immunoglobulin heavy variable 3-7; immunoglobulin heavy constant gamma 3 (G3m marker); immunoglobulin heavy variable 3-11 (gene/pseudogene); immunoglobulin heavy variable 4-31; immunoglobulin heavy locus |
| 3507 | IGH@ | Homo sapiens | immunoglobulin heavy constant gamma 1 (G1m marker); immunoglobulin heavy constant mu; immunoglobulin heavy variable 3-7; immunoglobulin heavy constant gamma 3 (G3m marker); immunoglobulin heavy variable 3-11 (gene/pseudogene); immunoglobulin heavy variable 4-31; immunoglobulin heavy locus |
| 3507 | IGHM | Homo sapiens | immunoglobulin heavy constant gamma 1 (G1m marker); immunoglobulin heavy constant mu; immunoglobulin heavy variable 3-7; immunoglobulin heavy constant gamma 3 (G3m marker); immunoglobulin heavy variable 3-11 (gene/pseudogene); immunoglobulin heavy variable 4-31; immunoglobulin heavy locus |
| 3507 | ighv4-31 | Homo sapiens | immunoglobulin heavy constant gamma 1 (G1m marker); immunoglobulin heavy constant mu; immunoglobulin heavy variable 3-7; immunoglobulin heavy constant gamma 3 (G3m marker); immunoglobulin heavy variable 3-11 (gene/pseudogene); immunoglobulin heavy variable 4-31; immunoglobulin heavy locus |
| 26105 | DKFZP434C153 | Homo sapiens | DKFZP434C153 protein |
| 3178 | LOC644037 | Homo sapiens | heterogeneous nuclear ribonucleoprotein A1-like 3; similar to heterogeneous nuclear ribonucleoprotein A1; heterogeneous nuclear ribonucleoprotein A1 pseudogene 2; heterogeneous nuclear ribonucleoprotein A1; heterogeneous nuclear ribonucleoprotein A1 pseudogene |
| 3178 | LOC645691 | Homo sapiens | heterogeneous nuclear ribonucleoprotein A1-like 3; similar to heterogeneous nuclear ribonucleoprotein A1; heterogeneous nuclear ribonucleoprotein A1 pseudogene 2; heterogeneous nuclear ribonucleoprotein A1; heterogeneous nuclear ribonucleoprotein A1 pseudogene |
| 3178 | HNRPA1L-2 | Homo sapiens | heterogeneous nuclear ribonucleoprotein A1-like 3; similar to heterogeneous nuclear ribonucleoprotein A1; heterogeneous nuclear ribonucleoprotein A1 pseudogene 2; heterogeneous nuclear ribonucleoprotein A1; heterogeneous nuclear ribonucleoprotein A1 pseudogene |
| 3178 | LOC728643 | Homo sapiens | heterogeneous nuclear ribonucleoprotein A1-like 3; similar to heterogeneous nuclear ribonucleoprotein A1; heterogeneous nuclear ribonucleoprotein A1 pseudogene 2; heterogeneous nuclear ribonucleoprotein A1; heterogeneous nuclear ribonucleoprotein A1 pseudogene |

| | | | |
|---|---|---|---|
| 3178 | HNRNPA1P2 | Homo sapiens | heterogeneous nuclear ribonucleoprotein A1-like 3; similar to heterogeneous nuclear ribonucleoprotein A1; heterogeneous nuclear ribonucleoprotein A1 pseudogene 2; heterogeneous nuclear ribonucleoprotein A1; heterogeneous nuclear ribonucleoprotein A1 pseudogene |
| 3178 | HNRNPA1 | Homo sapiens | heterogeneous nuclear ribonucleoprotein A1-like 3; similar to heterogeneous nuclear ribonucleoprotein A1; heterogeneous nuclear ribonucleoprotein A1 pseudogene 2; heterogeneous nuclear ribonucleoprotein A1; heterogeneous nuclear ribonucleoprotein A1 pseudogene |
| 3178 | HNRPA1L3 | Homo sapiens | heterogeneous nuclear ribonucleoprotein A1-like 3; similar to heterogeneous nuclear ribonucleoprotein A1; heterogeneous nuclear ribonucleoprotein A1 pseudogene 2; heterogeneous nuclear ribonucleoprotein A1; heterogeneous nuclear ribonucleoprotein A1 pseudogene |
| 2528 | FUT6 | Homo sapiens | fucosyltransferase 6 (alpha (1,3) fucosyltransferase) |
| 5460 | pou5f1 | Homo sapiens | POU class 5 homeobox 1 |
| 284443 | ZNF493 | Homo sapiens | zinc finger protein 493 |
| 3554 | Il1r1 | Homo sapiens | interleukin 1 receptor, type I |
| 2733 | GLE1 | Homo sapiens | GLE1 RNA export mediator homolog (yeast) |
| 3275 | PRMT2 | Homo sapiens | protein arginine methyltransferase 2 |
| 4137 | mapt | Homo sapiens | microtubule-associated protein tau |
| 1017 | Cdk2 | Homo sapiens | cyclin-dependent kinase 2 |
| 5902 | RanBP1 | Homo sapiens | similar to RAN binding protein 1; RAN binding protein 1 |
| 5902 | LOC389842 | Homo sapiens | similar to RAN binding protein 1; RAN binding protein 1 |
| 5902 | LOC727803 | Homo sapiens | similar to RAN binding protein 1; RAN binding protein 1 |
| 355 | Fas | Homo sapiens | Fas (TNF receptor superfamily, member 6) |
| 358 | aqp1 | Homo sapiens | aquaporin 1 (Colton blood group) |
| 55341 | lsg1 | Homo sapiens | large subunit GTPase 1 homolog (S. cerevisiae) |
| 5298 | PI4KB | Homo sapiens | phosphatidylinositol 4-kinase, catalytic, beta |
| 2035 | epb41 | Homo sapiens | erythrocyte membrane protein band 4.1 (elliptocytosis 1, RH-linked) |
| 65220 | nadK | Homo sapiens | NAD kinase |
| 1209 | CLPTM1 | Homo sapiens | cleft lip and palate associated transmembrane protein 1 |
